# IMPROVING LONGITUDINAL STUDIES OF AGING IN GEOSPATIAL HEALTH: AN AGENDA

**DOI:** 10.1093/geroni/igad104.0020

**Published:** 2023-12-21

**Authors:** Michael Desjardins, Kyle Moored, Emily Murray, Gergő Baranyi, Matthew Hobbs, Sarah Curtis, Michelle Carlson

**Affiliations:** Johns Hopkins Bloomberg School of Public Health, Baltimore, Maryland, United States; Johns Hopkins Bloomberg School of Public Health, Baltimore, Maryland, United States; University College London, London, England, United Kingdom; University of Edinburgh, Edinburgh, Scotland, United Kingdom; University of Canterbury, Christchurch, Canterbury, New Zealand; Durham University, Durham, England, United Kingdom; Johns Hopkins Bloomberg School of Public Health, Baltimore, Maryland, United States

## Abstract

Recent decades have seen a vast demographic shift in ageing populations worldwide, where over 1.4 billion people will be aged 60 years and older by 2030. Given specific populations may be more vulnerable to medical conditions with ageing, identifying high-risk locations are essential for allocating resources and promoting healthy ageing to mitigate population-level financial- and health-associated burdens. All aspects of public health research on ageing require longitudinal analyses to fully capture the dynamics of outcomes and risk factors such as human mobility, climate change, non-communicable diseases, and endemic, emerging, and re-emerging infectious diseases. Incorporating geospatial approaches in longitudinal health research can facilitate targeted interventions and improve public health policy and decision-making. Furthermore, geospatial approaches can identify where at-risk populations are located and what influences disease risk and exposure, particularly the ‘wider determinants’ of health across the lifecourse. However, studies in geospatial health are often limited to spatial and temporal cross sections. This generates uncertainty in the timing of exposures and behaviors. Geospatial longitudinal studies can better capture spatiotemporal dynamics of ageing. Here, we outline a research agenda, including key challenges and opportunities of working with longitudinal geospatial health data. Examples include accounting for residential and human mobility, recruiting new birth cohorts, geoimputation, international and interdisciplinary collaborations, spatial lifecourse studies, and qualitative and mixed-methods approaches. As a proof of concept, we present several case studies that better capture longitudinal exposures for the Cardiovascular Health Study cohort, which previously incorporated erroneous spatial indicators of neighborhood-level exposures across baseline and follow-up measures.

